# Gardening activity and its relationship to mental health: Understudied and untapped in low-and middle-income countries

**DOI:** 10.1016/j.pmedr.2022.101946

**Published:** 2022-08-08

**Authors:** Herbert E. Ainamani, Nolbert Gumisiriza, Wilson M. Bamwerinde, Godfrey Z. Rukundo

**Affiliations:** a Department of Mental Health, Kabale University School of Medicine, P. O. Box 317, Kabale, Uganda; b Department of Mental Health, Kabale University School of Medicine, Kabale, Uganda; c Department of Environment and Natural Resources, Kabale University, Kabale, Uganda; d Department of Psychiatry, Mbarara University of Science and Technology, Mbarara, Uganda

**Keywords:** Anxiety, Depression, Gardening, Mental health, Physical activity, Uganda, Sub-Saharan Africa

## Abstract

There is increasing awareness among researchers and health practitioners from high income countries about the potential mental health benefits of participating in gardening activities and spending substantial time in green spaces. However, this phenomenon is not well established in low- and middle-income countries. In this commentary, we discuss the evidence base surrounding the potential mental health benefits of participating in gardening activity and spending substantial time in a green space. We hope to stimulate discourse about incorporating these activities into mental health prevention in low- and middle-income countries.

## Highlights

• Participating in gardening activity may posses psycho-therapeutic elements.
• Participating in gardening improves mental health and enhences psychological well being.
• Participting in gardening seems to prevent mental health problems accross all ages.

## 1 Background

There is an increase in the number of people with mental health problems resulting from forced migration, domestic violence, chronic illnesses, caregiving burden, and other environmental stressors in low- and middle-income countries (LAMICs) (Patel, 2007).The mental health challenges are further exacerbated by food and water insecurity (Perkins et al., 2018) and under-resourced mental health systems (Molodynski, Cusack, & Nixon, 2017).This increasing burden of mental health problems will require innovations in the provision of low-threshold and culturally-relevant mental health interventions. Studies from high-income countries have suggested mental health benefits accruing from involvement in various forms of gardening (Clatworthy et al., 2013, Thompson, 2018). In Addition, other studies have cited mental health benefits of gardening and improvement in dementia symptoms (Gonzalez and Kirkevold, 2014, Murroni et al., 2021) and school children self-regulation (Weeland et al., 2019). However, these potential benefits are not well understood by mental health practitioners, researchers and policy makers from low- and middle-income countries. In this commentary, we highlight the potential benefits of participating in gardening activity and the reductions in symptoms of mental health problems based on the available litraure.

## 2 Gardening as a therapeutic intervention

Therapeutic benefits of participating in gardening have been widely recognized in high income countries (Detweiler et al., 2012; Schmutz, Lennartsson, Williams, Devereaux, & Davies, 2014). These benefits include high quality of life, sleep improvement, increased hope,happiness, reduction of symptoms of depression, stress, and anxiety (Ainamani et al., 2021).

Consistent with literature, ameta-analysis of the health effects of gardening and horticulture revealed a wide range of health benefits including reductions in body mass index, and increases in life satisfaction, quality of life and self-esteem (Soga, Gaston, & Yamaura, 2016). A systematic review that assessed the effectiveness of farm-based interventions for people with psychiatric problems recommended that farm-based interventions should be included in standard mental health treatment packages (Iancu et al., 2015). Another body of literature has shown that spending significant time in green space and caring for crops offers psycho- therapeutic benefits (Hassan et al., 2018, Thompson, 2018). All the above studies have emphasized the potential benefits of positive moods and mental wellbeing by participating in gardening or even observing nature.

## 3 Gardening for food security and mental health benefits

In Africa and other LAMICs, having a garden or participation in gardening is associated with an increase in food security and nutrition. On the other hand, having no garden is associated with food insecurity which has been linked to mental health distress and suicidal ideation (Sweetland et al., 2019). A study in rural Uganda,found a link between food insecurity and depression among both men and women (Perkins et al., 2018). Other studies have identified feelings of helplessness, shame, suffering, and humiliation as a centralaspect of the experience of food insecurity (Coates et al., 2006, Hamelin et al., 2002). Given these widespread benefits resulting from participating in gardening, clinicians should consider gardens as an important and promising health intervention.

## 4 Gardening as a physical activity for mental health benefits

Involvement in gardening presents various elements of physical exercise that have been shown to reduce an individual’s perception of stress and to improve overall mental health (Ghanbari et al., 2015, Soga et al., 2016). In addition, other studies have described participating in exercise activities as a useful intervention for reducing mental disorders (Powers et al., 2015, Rosenbaum et al., 2015). An earlier study by Fetzner and Asmundson (Fetzner & Asmundson, 2015) found clinically significant improvement and reduction in symptoms of PTSD after subjecting participants to an exercise activity. Furthermore, previous research has shown significantly higher reductions in depression, anxiety, and stress-symptom severity in patients who participated in an exercise treatment compared to controls (Powers et al., 2015, Rosenbaum et al., 2015). It is not surprising therefore,that participating in gardening activity has increasingly been recognized as a mental health treatment intervention (Pels & Kleinert, 2016).

## 5 Participating in gardening as a social activity

In consonance with the above literature, more evidence suggests that individuals with strong social support are likely to have low levels of symptoms of mental health problems (Gellert et al., 2018, Sugiyama et al., 2008, Tsai et al., 2012). In many settings, participation in gardening is more of a community activity and has elements of social support groups that will consequently accrue into psycho-social support (Lucke et al., 2019, Scott et al., 2020, Veen et al., 2016).Other studies continue to show that gardening provides opportunities to interact with family members and other community members which is likely to forge and reinforce social cohesion, community networks, and sense of community membership that improves general mental well being (Carney et al., 2012, Nanama and Frongillo, 2012).This kind of arrangement provides an opportunity for being together as individuals watch over crops and gardens which is likely to improve individuals’ self-esteem, teamwork, socia interaction, planning, problem solving and coping skills (Yeh and Liu, 2003, Zhao et al., 2013). Similarly, other studies have indicated that community gardens show great social support for people suffering from a range of mental disorders including alcohol use disorders (Carney et al., 2012, Schmutz et al., 2014). We therefore urgue that participation in gardening strengthens a sense of love of nature, community cohesion and social support which is very critical in the prevention and treatment of mental health problems.

## 6 Gardening as leisure activity

Where as gardening in LAMICs is done for the sake of livelihoods, in high-income countries gardening interventions are being deployed as leisure-time activities (Dunnett and Qasim, 2000, Ottosson and Grahn, 2005). For example, many people cultivate flowers, or engage in gardening for the calming activity that is internally pleasing(Cheng and Pegg, 2016, Wilkinson, 2003). In line with literature, a study that surveyed 397 participants in Southern England on the motivation for participating in a gardening activity found out that many of the participants enthusiastically participated in gardening activity as a hobby while others thought that a visit to a garden was quite enjoyable (Fox, 2017). Another study that randomly examined 6813 on their leisure time physical activities found out that (65%) of their participats endorsed gardening as the most popular leisure time activity (Rowinski, Dabrowski, & Kostka, 2015). Results from 433 older adults who were recruited for a gardening activity in Australia indicated that more than half of their participants obtained high levels of leisure relaxation, psychological and physiological well-being (Cheng et al., 2010).Owing to the differences in literature between the primary motivation for participating in gardening activity in LAMICs and high-income countries, it is important for this area of research to be explored in a setting where the primary function of gardening is for subsistence living.

## 7 Conclusions, recommendations and limitations

Our commentary provides evidence from literature that participating in gardening activity is psychologically therapeutic, improves food security and physical health. We propose that clinicians, researchers and policy makers consider participating in gardening activity as a potential mental health preventive intervention for people of all ages. Clinicians and other health care providers should encourage their patients to have small gardens in their yards or homesteads, create green space with trees and flowers around their homes. Gardens and green spaces should be planted around the hospitals and other medical centers. In addition, hospital wards should have windows that allow patients to view natural scenes and trees outside the buildings. Although gardening activity seems to be a promising tool for promoting mental health across all ages, limitations such as; lack of time, physical difficulties related to gardening activity especially for the elderly, lack of knowledge related to gardening activities, weather conditions and land pressure should be put into consideration.

## Declaration of Competing Interest

The authors declare that they have no known competing financial interests or personal relationships that could have appeared to influence the work reported in this paper.

## Data Availability

No data was used for the research described in the article.
